# Effects of comprehensive nursing intervention based on self-disclosure on improving alexithymia in elder patients with coronary heart disease

**DOI:** 10.1186/s12912-022-01006-w

**Published:** 2022-08-05

**Authors:** Weixin Zhang, Haili Zhang

**Affiliations:** Nursing School of Qiqihar Medical University, No 333, BuKui North Street, Jianhua District, Heilongjiang Province 161006 Qiqihar City, China

**Keywords:** Comprehensive nursing intervention, Alexithymia, Coronary heart disease, Recurrence, Physical activity

## Abstract

**Objectives:**

Patients with coronary heart disease (CHD) experience stress and suffer from the risk of recurrence and death. Comprehensive nursing intervention based on self-disclosure (CNISD) is an interdisciplinary service and an effective approach to care that improves quality of life and alleviates suffering for patients with CHD. The purpose of this study was to analyze the effects of CNISD on alexithymia in patients with CHD.

**Methods:**

A total of 1088 patients with CHD were recruited and received CNISD (*n* = 540) and usual care (*n* = 548). The quality of life, alexithymia, four statutory health insurance funds, recurrence, mortality, and satisfaction was compared in patients with CHD between CNISD and usual care group.

**Results:**

Outcomes showed that CNISD improved sleep quality and quality of life, increased physical activity, reduced the hospital anxiety and depression scale in patients with CHD compared to usual care. Recurrence and mortality of patients with CHD were markedly improved by CNISD compared to patients with CHD in usual care group.

**Conclusions:**

In conclusion, data in this study indicate that CNISD presents benefits in improving quality of life, physical activity, anxiety, depression, recurrence, and mortality for patients with CHD.

## Introduction

Coronary heart disease (CHD) is one of the leading causes of morbidity and mortality worldwide [1]. Although in China, CHD is the prime cause of mortality, the disease burden is now rising due to risk factors like hypertension, dyslipidemia, obesity, diabetes, smoking, unreasonable diet, lack of physical activity, excessive alcohol consumption, etc. [2]. Since cardiac rehabilitation is considered an effective modality to curb further disease progression, CHD patients frequently receive the guidance provided on healthy lifestyle changes regarding physical activity, a healthy diet, and nursing during rehabilitation in the hospital [3]. However, due to low emotional clarity, most of CHD patients develop symptoms of alexithymia, anxiety, and depression [4]. Alexithymia is defined as the disrupted emotional awareness, presents in a range of psychiatric and neurological disorders, and has a deleterious impact on functional outcomes and treatment response [5]. Alexithymia is a personality trait characterized by three dimensions: difficulty identifying feelings (DIF), difficulty describing feelings (DDF), and externally oriented thinking (EOT) [6, 7]. Alexithymia may also increase anxiety, depression, and stress, which can later become a predisposing factor to poor health and impaired quality of life along with inadequate social support [8].

Clinically, comprehensive nursing intervention is widely recognized as an effective approach to prevent the progression of patients with CHD [9]. Prompt nursing interventions can allow quality interactions between the patients and staff that can solve the quality of life and social problems [10]. Additionally, nursing intervention reduces anxiety and decrease the possibility of an acute cardiac event, which provides CHD patients with appropriate strategies for managing symptoms [11]. Furthermore, maintaining the quality of nursing intervention demonstrates a beneficial impact on secondary prevention in patients with coronary artery disease (CAD) or heart failure [12]. Therefore, it is crucial to investigate the effects of a comprehensive nursing intervention based on self-disclosure (CNISD) on alexithymia in elderly patients with CHD.

This study aimed to analyze the effect of CNISD on alexithymia in elder patients with CHD. The efficacy between CNISD and usual nursing was compared in improving alexithymia, anxiety, depression, stress, and quality of life in elderly CHD patients.

## Materials and methods

### Study design

This was a first analysis of data from a single center, randomized phase I clinical trial performed at Nursing School of Qiqihar Medical University. CHD patients were recruited between April 2017 and June 2019.

### Subjects

Patients with (*n* = 1088) were recruited from the Nursing School of Qiqihar Medical University (Qiqihar, China). Patients were diagnosed as CHD according to Diagnostic criteria for coronary heart disease [13]. All patients had been examined by three cardiologists, who had confirmed the diagnosis as coronary heart disease. Patients with CHD were randomly received post-operative CNISD (*n* = 540) and post-operative usual care (*n* = 548) within 3 months of the study. Cronbach’s alpha was used to estimate internal consistency reliability between two groups. Inclusion criteria: (1) age more than 60 years; (2) CHD patients. Exclusion criteria: (1) Patients with surgical or percutaneous revascularization; (2) major cardiac arrhythmia or use of a pacemaker or implantable cardioverter defibrillator; (3) major psychiatric disorder, cognitive impairment, pregnancy women.

### CNISD

Patients in the usual group received routine nursing. Usual nursing included diet instructions, nursing evaluation and drug dose reminder, etc. The CNISD project included usual care, the most common complications or adverse events in the care of CHD patients, enhanced preoperative care, enhanced post-operative care and discharge health guidance for all CHD patients based on self-disclosure. The most common complications included pressure sores, pain, anxiety, and risk factors leading to the above complications or adverse events. Enhanced preoperative care included understanding patients’ confidence, paying special attention to patients with other medical histories, evaluation of the patient’s disease status, preparing for disease prevention, arrangement of rest on time, preformation of muscle contraction exercise, instructing patients to learn sputum, defecation, and turning over in the bed. Post-operative care included observation the changes in the patient’s vital signs, the types of pathogens, conducting exercise guidance, evaluating the recovery of the CHD patients, guiding the diet care, and discharging health guidance.

### Measurements

Quality of life (QoL) of CHD patients was accessed using health-related to quality of life (WHOQOL-26) [14]. The Toronto Alexithymia Scale-20 items (TAS-20) was used to assess alexithymia of CHD patients, which has a three-factor structure based on the subscales differential item functioning (DIF), difficulty describing feelings (DDF), and externally oriented thinking (EOT) [15]. The Hospital Anxiety and Depression Scale (HADS) was used to calculate symptoms of depression (HADS-D) and anxiety (HADS-A) [16]. Sleep quality, sleep score and sleep duration was recorded during experiments in all CHD patients as described previously [17]. Stress scale of CHD patients was analyzed Multiple Scale Perceived Social Support (MSPSS-12). Physical activity of CHD patients was accessed using The ActiGraph GT3X + (*ActiGraph*, Pensacola, Florida, VS) and analyzed using the ActiGraph software (Version ActiLife 6.8). Physical activity was calculated into average total activity counts per hour (TAC/h) to compare physical activity between CNISD and usual care. Satisfaction of CHD patients in CNISD and usual care was analyzed using general satisfaction score as described previously [18]. Recurrence was recorded when patients had CAD symptoms [19].

### Statistical analysis

R-software (version 3.2.5, The R Foundation, Vienna, Austria) was used to analyze the data. All continuous variables are expressed as mean ± SD and for some categorical as numbers and percentages. Paired samples *t*-test was used to compare quantitative variables. Independent samples *t*-test was used for intergroup comparisons. Qualitative variables were compared with the chi-square test. Statistical significance was set at *p* < 0.05.

## Results

### Characteristic of patients with coronary heart disease

**Fig. 1:**
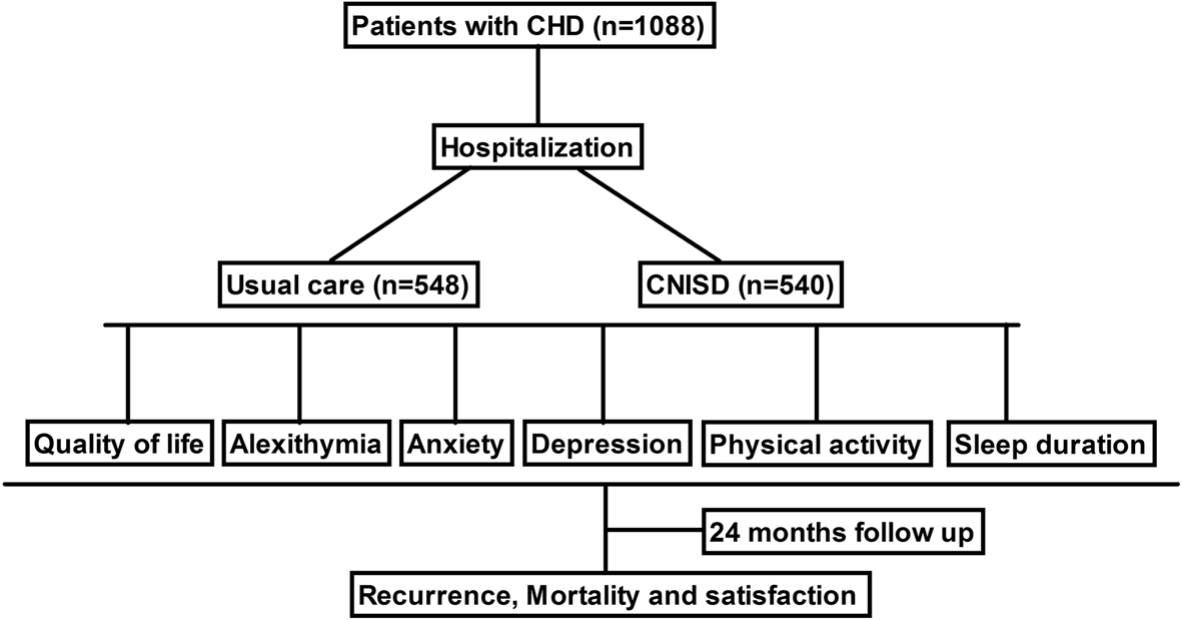
Study flow chart of CHD patients

The main characteristics of CHD patients are summarized in Table 1. A total of 1088 patients with CHD were recruited in Qiqihar Medical University between May 2017 and June 2019. CHD patients received CNISD (*n* = 540) and usual care (*n* = 548). The study design is shown in Fig. 1. There were no significant differences of quality of life, alexithymia, depression scale and anxiety between CNISD and usual care group. Analysis of data showed that Cronbach’s alpha value was 0.842, which indicated a good internal consistency reliability between two groups.

**Table 1:** Characteristics of patients with mild coronary heart disease

	Usual care	CNISD	*P* value
Coronary artery disease	540 (49.63%)	548 (50.37%)	0.93
Gender (male/female)	260/280	256/292	0.84
Age (years)	60 ± 8	58 ± 10	0.96
BMI (kg/m^2^)	26.42 ± 3.42	26.26 ± 3.56	0.80
Smoking history	345 (63.0%)	336 (62.2%)	0.75
History of hypertension	285 (52.0%)	280 (51.9%)	0.85
Total cholesterol (mmol/L)	3.92 ± 0.80	3.90 ± 0.84	0.97
Triglycerids (mmol/L)	1.40 ± 0.62	1.43 ± 0.68	0.84
HDL-c (mmol/L)	1.66 ± 0.75	1.70 ± 0.80	0.87
LDL-c (mmol/L)	3.05 ± 0.80	3.20 ± 0.90	0.90
hs-CRP (mg/L)	6.12 ± 0.81	6.20 ± 0.75	0.88

Data are expressed as mean ± SD or n (%)

### Effects of CNISD on quality of life, alexithymia, anxiety, and depression in CHD patients

**Fig. 2:**
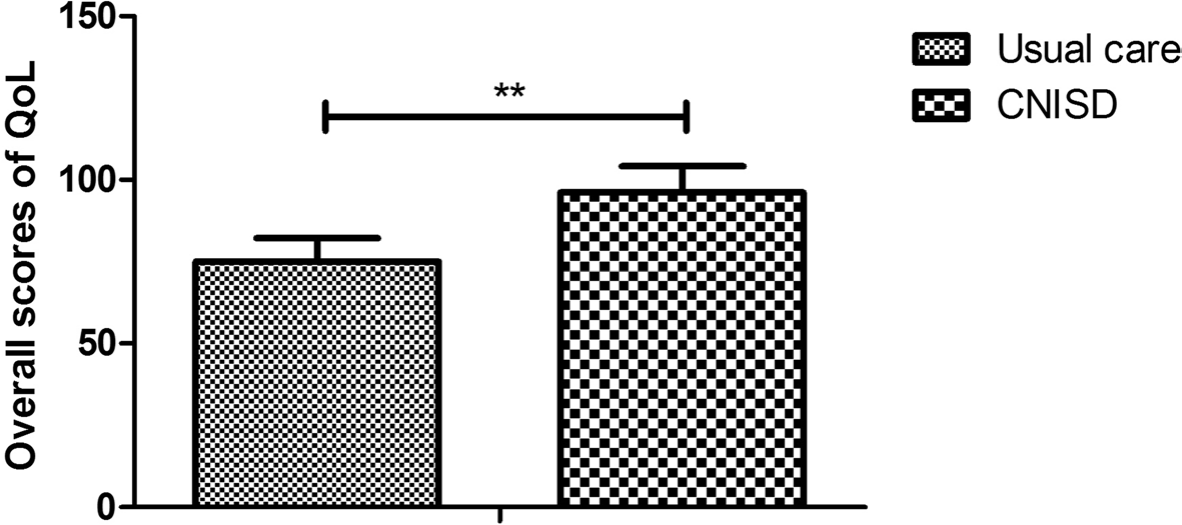
Effect of CNISD on quality of life in CHD patients between CNISD and usual care group. **p < 0.01 vs. usual care

There were several differences in patients with CHD between CNISD and usual care group. Outcomes showed that CNISD significantly increased the quality of life of CHD patients compared to usual care (Fig. 2, Cronbach’s alpha was 0.88). CNISD decreased alexithymia of CHD patients compared to usual care (Table 2, Cronbach’s alpha was 0.82). Date revealed that CNISD markedly improved DIF, DDF, and EOT of CHD patients compared to patients in usual care group (Table 2, Cronbach’s alpha was 0.80). The number of CHD patients with alexithymia were also decreased by CNISD compared to patients in usual care group. Notably, CNISD markedly improved anxiety and depression of CHD patients compared to usual care (Table 2, Cronbach’s alpha was 0.78).

**Table 2:** Effect of CNISD on quality of life, alexithymia, anxiety and depression in CHD patients

	Usual care	CNISD	*P* value
Alexithymia			
DIF	160 (14.7%)	60 (5.5%)	0.003
DDF	108 (9.9%)	80 (7.4%)	0.036
EOT	280 (25.7%)	320 (29.4%)	0.024
HADS-A scores (anxiety)	12 ± 3	8 ± 2	0.037
HADS-D scores (depression)	50 ± 10	12 ± 3	0.001

Data are expressed as mean ± SD or n (%)

### Effects of CNISD on physical activity and sleep in CHD patients

**Fig. 3:**
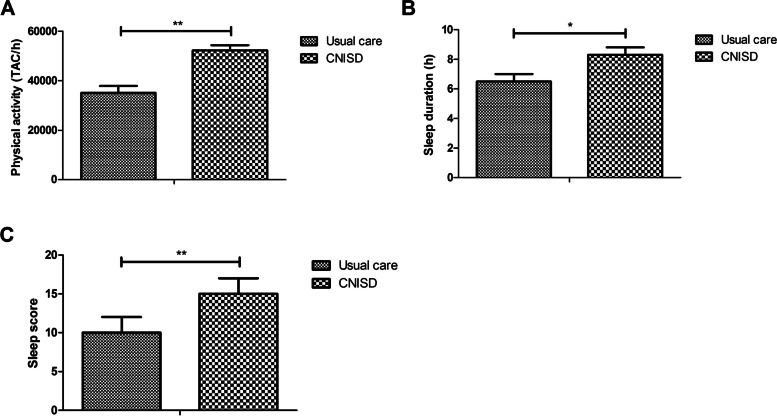
Effect of CNISD on physical activity and sleep quality in CHD patients between CNISD and usual care group. **A** Effect of CNISD on physical activity of CHD patients between CNISD and usual care group. **B** Effect of CNISD on sleep duration of CHD patients between CNISD and usual care group. **C** Effect of CNISD on sleep score of CHD patients between CNISD and usual care group. TAC/h, average Total Activity Counts per hour. *p < 0.05, **p < 0.01 vs. usual care

The improvements of physical activity and sleep were compared in CHD patients between CNISD and usual care group. Data showed that CNISD significantly improved physical activity of CHD patients compared to usual care (Fig. 3A; *p* < 0.01). CNISD increased sleep duration of CHD patients compared to those in usual care group (Fig. 3B; *p* < 0.05). The participants with long sleep duration (> 8 h/d) reached statistical significance in CNISD group compared to patients in usual care group (Table 3). CHD patients in CNISD group had higher sleep score than those in usual care group (Fig. 3C; *p* < 0.01).

**Table 3:** Effect of CNISD on sleep duration in CHD patients

	Usual care	CNISD	*P* value
≥ 8 h/day	260 (47.4%)	325 (60.1%)	0.0082
4–6 h/day	184 (33.6%)	172 (31.9%)	0.062
< 4 h/day	104 (19.0%)	43 (8.0%)	0.001

Data are expressed as n (%)

### Effects of CNISD on recurrence, mortality, and satisfaction in CHD patients

**Fig. 4:**
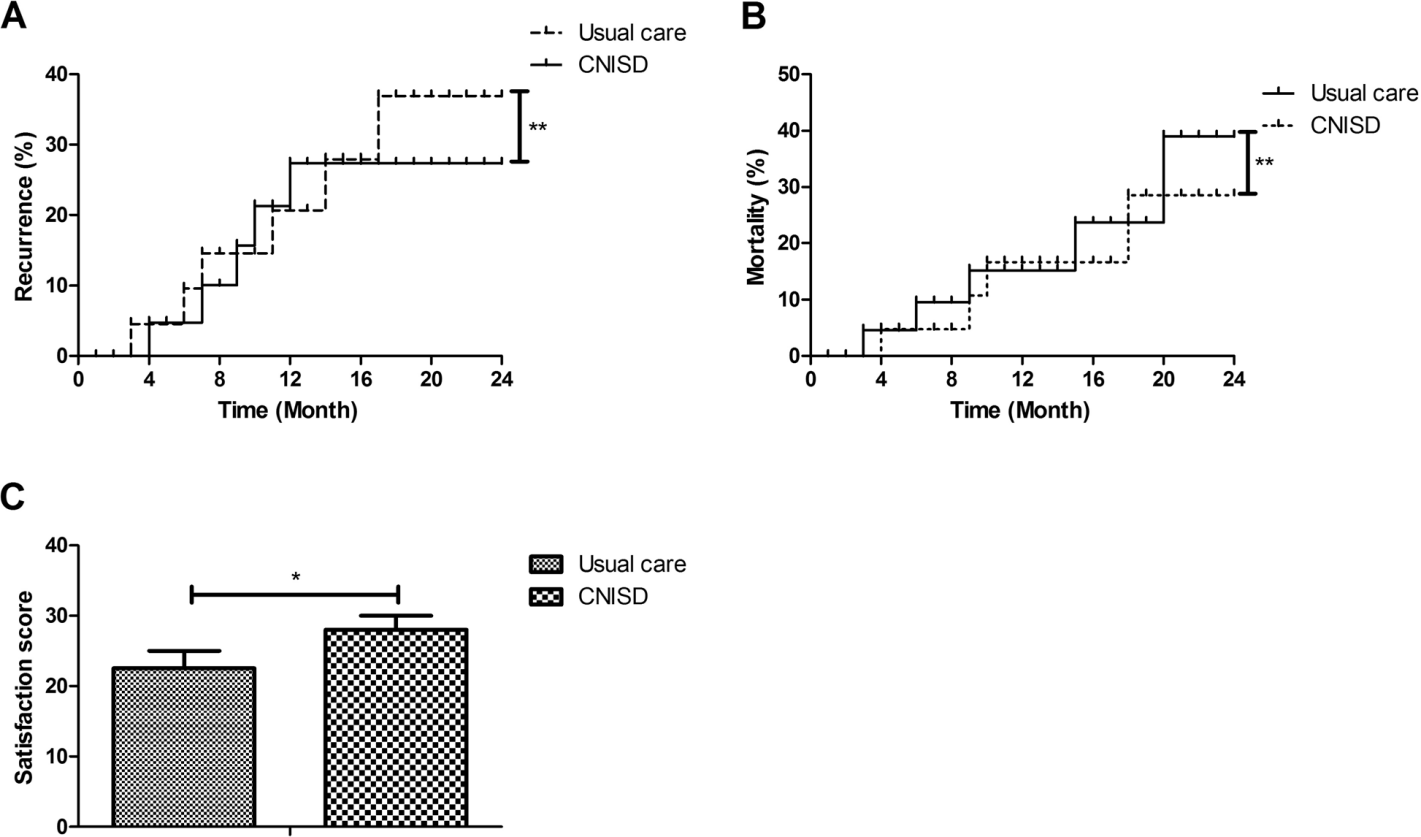
Effect of CNISD on recurrence, mortality, and satisfaction in CHD patients between CNISD and usual care group. **A** Recurrence of CHD patients between CNISD and usual care group. **B** Mortality of CHD patients between CNISD and usual care group. **C** Satisfaction score of CHD patients between CNISD and usual care group. *p < 0.05, **p < 0.01 vs. usual care

At the end of investigation, recurrence, mortality, and satisfaction were analyzed in CHD patients between the two groups. Data showed that CNISD decreased recurrence of CHD patients compared to usual care during 24-month follow up (Fig. 4A; *p* < 0.01). A lower mortality of CHD patients was observed in CNISD group compared to those in usual care group (Fig. 4B; *p* < 0.01). Statistical analysis showed that satisfaction score was higher in CHD patients in CNISD group than those in usual care group (Fig. 4C; *p* < 0.05). Cronbach’s alpha for global satisfaction scale was 0.87, demonstrating a good degree of internal consistency among the individual items. Table 4 showed that the number of satisfied CHD patients in CNISD group was higher than those patients in usual care group.

**Table 4:** Analysis of effect of CNISD on general satisfaction in CHD patients

	Usual care	CNISD	*P* value
Unsatisfied	185 (33.8%)	85 (15.7%)	0.006
Occasionally satisfied	146 (26.6%)	70 (13.0%)	0.008
Satisfied	217 (39.6%)	385 (71.3%)	0.001

Data are expressed as n (%)

## Discussion

Given that adherence to CHD patients’ guidelines in nursing is generally low, even when these guidelines predominantly comprise foundational recommendations, it is important to explore appropriate care plans to improve the quality of life, alexithymia, anxiety, and depression of CHD patients [20, 21]. A previous study provided an experimental basis for the clinical application of comprehensive nursing intervention in CHD patients [22]. This is the first cohort study to investigate the effects of CNISD on quality of life, alexithymia, anxiety, depression, physical activity, sleep, recurrence, mortality, and satisfaction in CHD patients. Our results reported that CNISD not only increased sleep quality, but also improved the quality of life, alexithymia, anxiety, and depression in CHD patients when compared to usual medical care. Because recurrence and mortality are two pivotal risk factors, our results suggest the importance of CNISD when developing strategies to decrease the recurrence and mortality of CHD patients.

Following improved survival rates in patients with CHD, the quality of life and its determinants have become increasingly prominent for obtaining positive patient outcomes [23]. Alexithymia is associated with the enhanced psychosocial burden of suffering CHD [24]. Our results suggested that CNISD increased the quality of life, decreased alexithymia, and enhanced the physical activity of CHD patients when compared to usual medical care. Depression, anxiety, and stress are strongly associated with CHD, antidepressants and psychotherapy can improve the control of mental disorders and quality of life and, in some cases, create a positive impact on the course of CHD [25]. Patients with CHD present poor physical activity that is inversely associated with mortality [26]. Outcomes in this study reported that the physical activity of CHD patients was significantly improved by CNISD, which further contributed to lower mortality. These data indicated that CNISD could decrease cardiovascular risk factors, which were related to self-disclosure on alexithymia in elder patients with CHD.

Few previous studies have reported that insomnia or short sleep duration increases the risk of CHD [17, 27]. On the contrary, another systematic review reported that sleeping for more than 8 h/d is associated with an increased risk of CHD [28]. Moreover, the effects of nursing interventions on sleep duration have not been fully investigated in CHD patients [29–31]. This is the first study to analyze the effects of CNISD on sleep quality, anxiety, and depression in CHD patients. Our results found that statistical significance was observed between CNISD and prognosis as compared to usual care. These data indicated that the association between a decrease in mortality and greater physical activity was stronger in the patients who revived CNISD. However, CNISD was only used in a minority of patients in China.

In conclusion, our study demonstrates the potential of CNISD in improving physical activity, sleep quality, quality of life, alexithymia, depression, and anxiety in patients with CHD. These data have implications for clinical nursing of CHD patients, suggesting CNISD may help patients with CHD avoid a high risk of recurrence and mortality. Additional large prospective studies are required to confirm the observed benefits of CNISD in CHD patients for improving the clinical outcomes.

## Data Availability

The datasets used and/or analyzed during the current study available from the corresponding author on reasonable request.
